# Polylactic acid: Is it everything the same?

**DOI:** 10.1111/jocd.16539

**Published:** 2024-08-20

**Authors:** Airá Novello Vilar, Vitoria Azulay, Estevão Vargas, Regina Casz Schechtman

**Affiliations:** ^1^ Institute of Dermatology Professor Rubem David Azulay Santa Casa de Misericórdia do Rio de Janeiro Rio de Janeiro Brazil

**Keywords:** collagen, histopathology, polylactic acid

To the Editor,

Poly‐L‐lactic acid (PLLA) is a biocompatible, biodegradable, and synthetic polymer implant that acts by stimulating an inflammatory response providing volume and neocollagenesis.^1^ It was first introduced for treatment to lipotrophic HIV patients in 2004 and since then, it has been used safely for soft‐tissue implants, vollumization, and stimulating collagen production.^2^

PLLA is supplied as lyophilized powder, carboxymethylcellulose, and nonpyrogenic mannitol that are mixed with distilled water in order to hydrate. PLLA microparticles, once injected, stimulate a foreign body inflammatory response with consequent fibroplasia and collagen type 1 production.^3^ At 3 months, there is an increase in collagen fibers, including collagen type III.

Although considered a safe treatment, it is not devoid of complications. One of the most frequent complications is the formation of persistent nodules. Histopathologically, these are characterized as foreign body granulomas containing multinucleated giant cells.^4^

Over recent years, there has been a significant increase in the use of PLLA, including the emergence of new brands on the market. It is important to keep in mind that although they are all PLLA, their composition is not the same. The objective of this study is to analyze microscopically the composition of pure product, in vitro, 1 day after dilution of two existing PLLA, Sculptra®, and Elleva®.

The comparative analysis of the commercial products Sculptra® and Elleva® by optical microscopy stained by HE demonstrates refringent crystals, more dispersed in Sculptra®, and more grouped in Elleva®. Alcian blue staining shows areas that stain positively (blue) in the Elleva® next to the crystals, suggesting that there are acidic mucins in the vehicle used; which was not observed in the same coloring for Sculptra® (Figures 1 and 2). Furthermore, Sculptra® crystals look like little needles and Elleva® are rounded and smaller.

**FIGURE 1:**
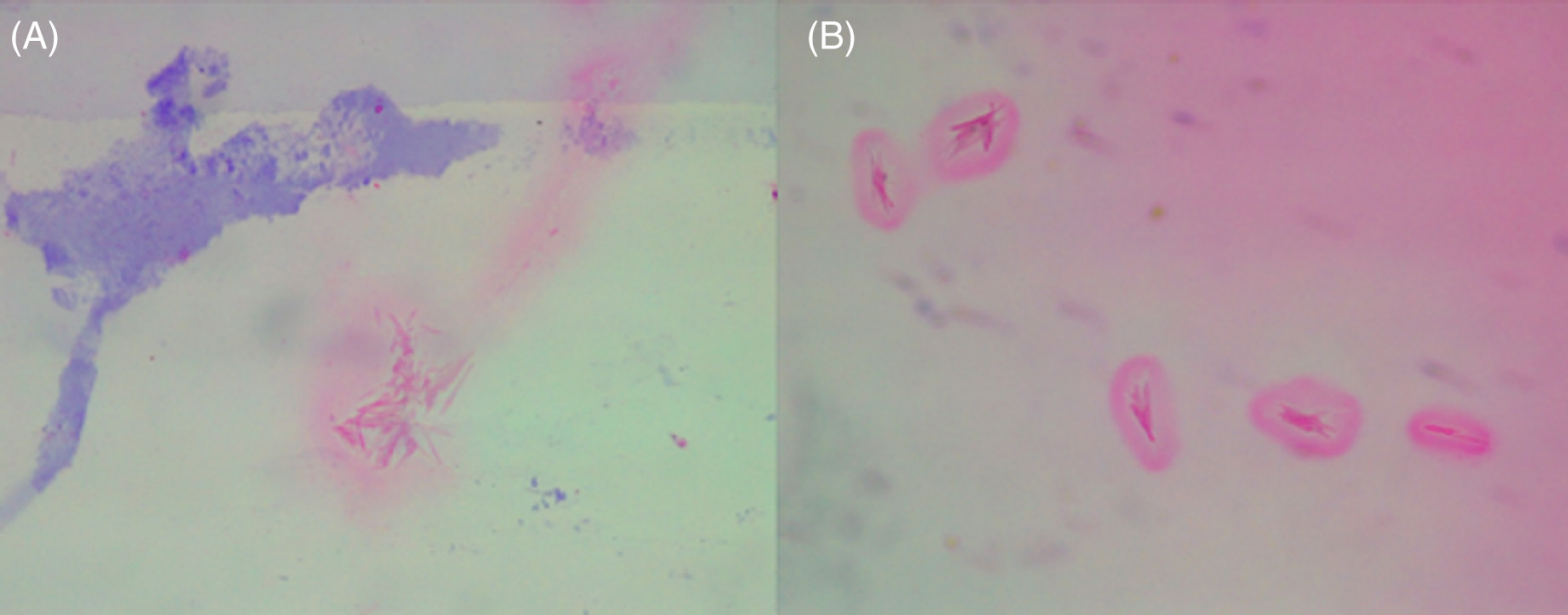
(A) Alcian blue, MO staining (optical microscopy) 10× of Elleva®: In the center, “grouped” crystals are evident that are refringent to polarizing microscopy. In the upper left corner in blue, material positively stained by the alcian blue technique (acid mucins). (B) Alcian blue staining, MO (optical microscopy) 10× of Sculptra®: Crystals throughout the microscopic field, surrounded by pink colored material (vehicle), which is not stained by the alcian blue technique.

**FIGURE 2:**
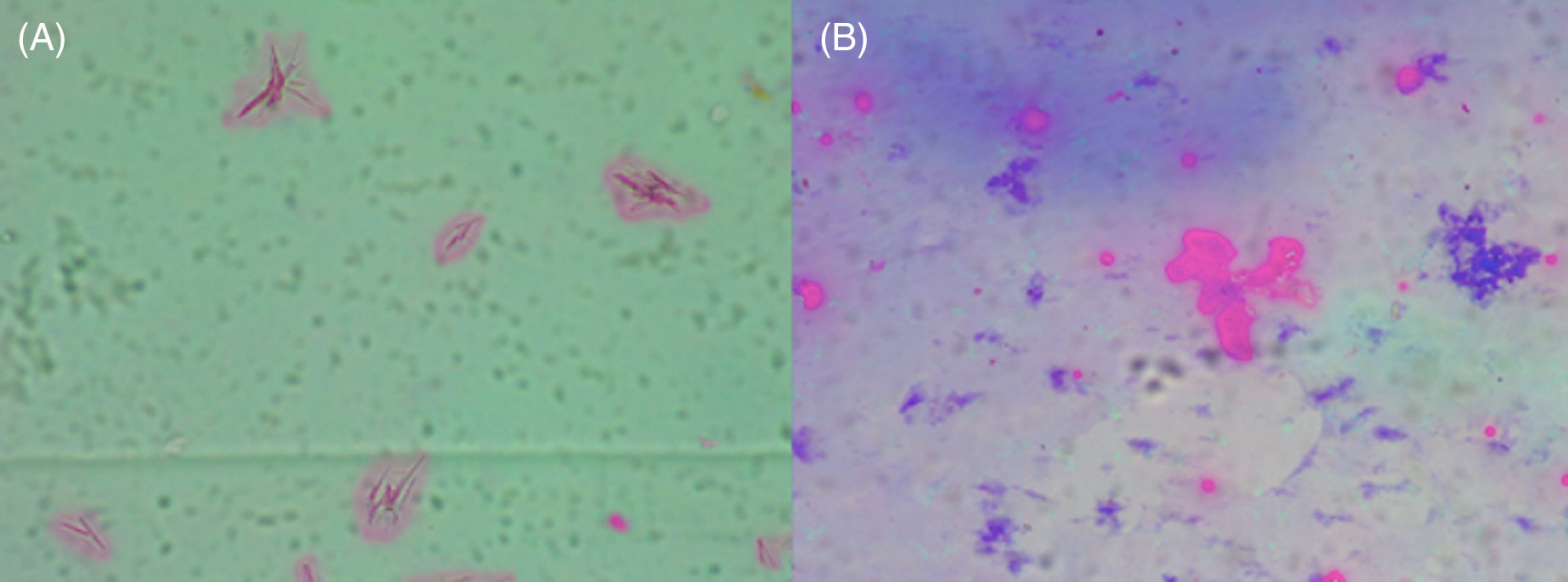
(A) Sculptra® polarized disperse. (B) Elleva® polarized: The crystals aggregate in the center and there is this material that are stained by Alcian Blue on the side.

In conclusion, there is no significant difference related to cost between both products. However, Sculptra® is a well‐known PLLA present on the market for 25 years, with few complications. A recent study showed that half of the complications arising from bio‐stimulators are attributed to Elleva®, with harder nodules and poorest resolution.^5^

Regarding ultrasound comparison, Elleva® showed strong acoustic shadowing on ultrasound, different from Sculptra®, showing that there is a failure of the sound bean to pass through it.^6^ Shadowing is also seen in fibrotic tissue, which may also explain Elleva® harder nodules.

Therefore, it is important to know the appearance of the product in order to differentiate them through a complication and provide appropriate treatment.

## AUTHOR CONTRIBUTIONS

A.N.—performed the research, designed the research study, and wrote the paper. V.A.—performed the research, designed the research study, and wrote the paper. E.V.—wrote the paper and analyzed the data. R.C.S.—contributed essential tools and analyzed the data. All authors have read and approved the final version of the manuscript.

## ETHICS STATEMENT

The study was approved by the ethical review board.

## Data Availability

The data that support the findings of this study are available from the corresponding author upon reasonable request.
